# The Nuka System of Care: improving health through ownership and relationships

**DOI:** 10.3402/ijch.v72i0.21118

**Published:** 2013-08-05

**Authors:** Katherine Gottlieb

**Affiliations:** Southcentral Foundation, Anchorage, AK, USA

**Keywords:** Alaska Native, wellness, self-determination, relationships, outreach, public health, quality improvement

## Abstract

Southcentral Foundation’s Nuka System of Care, based in Anchorage, Alaska, is a result of a customer-driven overhaul of what was previously a bureaucratic system centrally controlled by the Indian Health Service. Alaska Native people are in control as the “customer-owners” of this health care system. The vision and mission focus on physical, mental, emotional, and spiritual wellness and working together as a Native Community. Coupled with operational principles based on relationships, core concepts and key points, this framework has fostered an environment for creativity, innovation and continuous quality improvement. Alaska Native people have received national and international recognition for their work and have set high standards for performance excellence, community engagement, and overall impact on population health. In this article, the health care transformation led by Alaska Native people is described and the benefits and results of customer ownership and the relationship-based Nuka System of Care are discussed.

Southcentral Foundation is a non-profit health care organization serving more than 60,000 Alaska Native and American Indian people in Southcentral Alaska. It was established in 1982 under the tribal authority of Cook Inlet Region Inc. (CIRI), one of the Alaska Native regional corporations created by Congress in 1971 under the terms of the Alaska Native Claims Settlement Act. CIRI established Southcentral Foundation to improve the health and social conditions of Alaska Native people, enhance culture and empower individuals and families to take charge of their lives. Southcentral Foundation's “Nuka System of Care” is a term that describes the entire health care system created, managed and owned by Alaska Native people to achieve physical, mental, emotional and spiritual wellness. It is inclusive of all parts of the organization – including behavioral, dental, medical and traditional services – and all the systems, processes and departments supporting the service delivery.

## History

Over the last 3 decades, Southcentral Foundation's workforce has grown from fewer than 25 to more than 1,500 employees and the operating budget from $3 million to $210 million. This growth can be attributed in large part to a change in ownership of the Alaska Native health care system – from government control to “customer ownership.”

For 50 years, Alaska Native people in Southcentral Alaska received their health care as “beneficiaries” of the Indian Health Service's Native hospital. Employees were not able to be creative or innovative because it was a large, bureaucratic system centrally controlled from Washington, DC, 5,000 miles away. Patients waited weeks to get an appointment or accessed the system through the emergency room, and saw different providers each time. There was a disconnect between care for the mind and care for the body. Departments and programs acted independently. Patients were not happy and employees were not happy. Health statistics were bleak. Many patients left the Alaska Native system altogether to find better care (1). Then, in response to Alaska Native and American Indian people advocating for a voice in program planning and service delivery, Congress passed a federal law in favor of self-determination (2,3). This legislation opened the door for tribes to choose ownership over the entities delivering the services. The Alaska Native leadership of Southcentral Foundation also saw this as an opportunity for innovation – to completely redesign the tribal health care system in Southcentral Alaska based on Alaska Native values and needs. The administration and Southcentral Foundation partnered to survey the Native Community and find out what was desired.

By 1999, Alaska Native people were no longer “beneficiaries” of a government-run system, but, rather, chose to become self-determined “customers” and also “owners” of their tribally managed health care. This meant that Alaska Native people were no longer mere recipients of services, but, rather, in control of decision-making and administration. Along with this new customer-owner status came responsibilities to make informed choices on priorities for the health care system and to work to sustain it for future generations; what followed was a customer-driven overhaul of health care delivery, philosophy and values. As a result, Southcentral Foundation has today what is known as its Nuka System of Care. It addresses the challenges that health care systems around the world face – how to improve health care outcomes and customer satisfaction without skyrocketing costs.

## Shared vision and mission

Southcentral Foundation's vision is “A Native Community that enjoys physical, mental, emotional and spiritual wellness.” The organization is committed to doing more than just providing treatment and health education. Southcentral Foundation's barometer for success is whether the population served is able to truly experience multidimensional wellness, and if improvements in wellness are experienced from one generation to the next.

The mission statement emphasizes getting there by working with (not doing “to” or “for”) the Native Community. The aim is a Native Community that is renowned for being healthy. Southcentral Foundation measures its progress through a robust data collection effort, benchmarking with other high-performing health care organizations around the country and tracking health disparity data at the local, state and national levels.

Southcentral Foundation is intentional in the way it communicates its mission and vision to the community, workforce and customer-owners. The vision and mission provide guidance and consistency; there is a clear message and path to follow. All corporate, division, work unit, and individual goals and objectives flow out of the vision and mission's 3 “key points”: shared responsibility, commitment to quality and family wellness. This framework, established by the Alaska Native board of directors, keeps Southcentral Foundation's performance evaluation and improvement efforts focused on achievement of the vision and mission. The governing board, which is composed entirely of customer-owners, sets the direction and the president/CEO creates an environment that ensures the entire workforce can both stay the course and measure progress along the way. As a result, Southcentral Foundation's data analysis and tracking ties directly back into fulfillment of the vision and mission, and achievements are shared with stakeholders in a meaningful way. For example, under the corporate goal of “shared responsibility” there are 3 corporate objectives – one of which is “achieve excellence in customer-owner satisfaction.” Knowing that appointment access is a key driver of customer-owner satisfaction, departments created work plans and measurement targets around improving the availability of appointments. The data collection approach included tracking average appointment availability at 8:00 am daily, the “third next available appointment” less than 5 days out, as well as the medians and other subreports. These operational measures are available on a centralized “data mall” and are segmented to the appropriate level to support improvement of day-to-day work processes.

##

**Vision Statement**

A Native Community that enjoys physical, mental, emotional and spiritual wellness.

**Mission Statement**

Working together with the Native Community to achieve wellness through health and related services.

**Key Points**

***Shared Responsibility***

We value working together with the individual, the family, and the community. We strive to honor the dignity of every individual. We see the journey to wellness being traveled in shared responsibility and partnership with those for whom we provide services.

***Commitment to Quality***

We strive to provide the best services for the Native Community. We employ fully qualified staff in all positions and we commit ourselves to recruiting and training Native staff to meet this need. We structure our organization to optimize the skills and contributions of our staff.

***Family Wellness***

We value the family as the heart of the Native Community. We work to promote wellness that goes beyond absence of illness and prevention of disease. We encourage physical, mental, social, spiritual & economic wellness in the individual, the family, the community and the world in which we live.

## Service delivery

To achieve its vision, Southcentral Foundation provides a wide range of behavioral, dental, medical and community services. These services include primary care, both in outpatient and home settings; dentistry; outpatient behavioral health; residential behavioral health; traditional healing; complementary medicine; health education and more. In addition, Southcentral Foundation has administrative programs that support direct service delivery, including human resources, information technology, compliance, grants, public relations, finance, facilities and quality assurance.

In general, Southcentral Foundation's services are provided “prepaid,” based on legislative agreements and funding requirements, to members of 227 federally recognized Alaska Native tribes who live in Anchorage, the Matanuska-Susitna Valley and 55 rural Anchorage Service Unit villages. This 108,000-square-mile service area stretches about 2,000 miles from west to east, in a state that is nearly 3 times the size of Texas.

As significant numbers of Alaska Native people continue to migrate out of Alaska's rural areas to the urban centers (4), most customer-owners live in or near Anchorage, home of the Alaska Native Medical Center's 150-bed hospital and the Anchorage Native Primary Care Center, and other Southcentral Foundation owned and co-owned facilities and services. Care delivery mechanisms include ambulatory office visits, home visits, email and telephone visits, health information and education via classes and mixed media, inpatient hospital services, day and residential treatment, as well as consultation with and referral to higher levels of care. Southcentral Foundation also jointly owns and manages the Anchorage-based Alaska Native Medical Center with the Alaska Native Tribal Health Consortium (ANTHC). When advanced and complex care is required, Southcentral Foundation engages a seamless continuum of care by working in partnership with the tertiary and specialty Medical Services Division of ANTHC.

Southcentral Foundation also has experience in distance delivery of health care. Southcentral Foundation's clinical teams regularly travel to villages off the road system – accessible only by air or boat – to deliver family medicine, behavioral health, dental and optometry services. Where village clinics are in place, Southcentral Foundation clinicians also make use of electronic communication, including state-of-the-art telemedicine technology, to consult on assessment and treatment. In some cases, appropriate treatment requires Southcentral Foundation to bring customer-owners from the rural communities to Anchorage.

## Relationships

Southcentral Foundation's Nuka System of Care is based on what customer-owners really want – a primary focus on building and maintaining relationships.

Research findings have shown that relationship-based partnerships, over time, have the power to influence health outcomes (5–10). In the Nuka System of Care, one of the chief responsibilities of each provider is to work with customer-owners to establish trusting, accountable and long-term relationships. Relationships provide a better understanding of the context in which a customer lives. As a result, providers are in a better position to understand symptoms, answer questions, have meaningful conversations about risks and benefits, and work with each customer to make better health decisions. These basic principles are consistently put into practice by Southcentral Foundation's medical, behavioral, dental and traditional service providers.

However, the focus is not only on building relationships between providers and customer-owners. Southcentral Foundation's operational principles, which spell out “R-E-L-A-T-I-O-N-S-H-I-P-S,” influence everything from the strategic planning process to employee hiring practices, facility design, job progressions, information support, quality improvement, financing structures, work flow across boundaries and more.

Strong and effective relationships are necessary across the organization to accomplish goals, objectives and work plans. Building a culture of trust, based on relationships, encourages shared decision-making and supports innovation and creativity.

The organization's executive leaders role model relationship-building behaviors for the rest of the workforce, including sharing personal stories, inviting inquiry and questions, admitting mistakes and celebrating successes. A 3-day mandatory Core Concepts training, led by the president/CEO, helps employees understand how their relational styles impact others, how their experiences affect how they approach and build relationships, and how to articulate and respond to story in everyday work and life.

Southcentral Foundation also depends on relationships with national, regional and local partners. The focus is more on collaboration than competition. As a result, service gaps are identified and new collaborations emerge each year.

Over a decade of performance measurement data has shown that the relationship-based Nuka System of Care has effectively broken down barriers – including barriers of space, attitude, language and time – that previously stood in the way of better health and wellness.

##

**Operational Principles**

**R**elationships between the customer-owner, the family, and provider must be fostered and supported

**E**mphasis on wellness of the whole person, family, and community including physical, mental, emotional, and spiritual wellness

**L**ocations that are convenient for the customer-owner and create minimal stops for the customer-owner

**A**ccess is optimized and waiting times are limited

**T**ogether with the customer-owner as an active partner

**I**ntentional whole system design to maximize coordination and minimize duplication

**O**utcome and process measures to continuously evaluate and improve

**N**ot complicated but simple and easy to use

**S**ervices are financially sustainable and viable

**H**ub of the system is the family

**I**nterests of the customer-owner drive the system to determine what we do and how we do it

**P**opulation-based systems and services

**S**ervices and systems build on the strengths of Alaska Native cultures

**Core Concepts**

**W**ork together in relationship to learn and grow

**E**ncourage understanding

**L**isten with an open mind

**L**augh and enjoy humor throughout the day

**N**otice the dignity and value of ourselves and others

**E**ngage others with compassion

**S**hare our stories and our hearts

**S**trive to honor and respect ourselves and others

## Customer ownership

The shift to customer ownership, including the involvement of Alaska Native people in the design, implementation and control of their own programs, has produced dramatic changes in the delivery of health care services, in Alaska Native people's sense of self-efficacy, and ultimately, in health outcomes.

With customer-owners originating from more than 200 tribes in Alaska alone, Southcentral Foundation works in partnership with many different cultural groups. To ensure the organization is capturing feedback from this diverse customer base, it offers a range of options for customer-owners to be heard and responded to – some examples include personal interaction with staff, comment cards, special events, surveys, a 24-h telephone hotline and online form, focus groups and advisory committees.

Southcentral Foundation's board of directors and advisory boards are comprised solely of Alaska Native customer-owners, representing a number of different tribes. Customer-owners have also established careers at Southcentral Foundation in an increasing number of both clinical and non-clinical roles. The majority of the workforce is, in fact, Alaska Native and American Indian, including the long-time president/chief executive officer, 2 vice presidents, and over 60% of the organization's managers. Internship programs, succession planning and other workforce development initiatives are continuously grooming the next generation to take over paraprofessional, professional and leadership roles within the organization.

Alaska Native and American Indian employees also have an active role as members of Southcentral Foundation's 4 functional committees – process improvement, quality improvement, quality assurance and operations. The committees were created to be responsive to customer-owner feedback and move improvement initiatives and work plans forward without having to take ideas to the executive leadership team. The relationship-based operational principles are used to measure the alignment of any specific improvement idea. Any idea from an employee or customer-owner using the system can be put forward, and, if there is good alignment with the principles, an effort will be made to support testing that idea.

Before the Nuka System of Care, far too many Alaska Native people believed that they had no control or opportunity for input. This belief was conditioned over many decades of well-intended government-run health care that promoted the message “we will take care of you.” To reverse this took a concentrated effort and empowerment on many different levels. While the system is not perfect, there have been measurable improvements. For example, a recent yearlong survey asking customer-owners about their experiences in Southcentral Foundation's clinics showed that 98.5% of the respondents agreed with the following statement: “I was given the chance to provide input into decisions about my health care.” Another example – lower scores in the “Wait time to be seen by my provider” survey question initiated improvement efforts to make same-day access a priority.

The Nuka System of Care is a departure from “beneficiaries” or “patients” serving as mere recipients of tests, diagnoses, and pills. Instead, customer-owners actively share responsibility for the success of the health care system and for their family's health and wellness.

## Results

The keys to Southcentral Foundation's improvement journey and resulting success can be distilled down to: (a) customer ownership and (b) relationships. Health care leaders from around the world attend Southcentral Foundation's annual Nuka System of Care Conference to learn more about these approaches, including how they lead to the implementation of best practices such as organization-wide “advanced access,” utilization of data and measurement, integrated care teams and integration of behavioral health and traditional healing into primary care.

The relationship-based, customer-owned Nuka System of Care has helped Southcentral Foundation outperform many known health care systems. It works because Southcentral Foundation redesigned the entire health care system based on the wants and wishes of its customer-owners, and, in doing so, empowered those receiving the services to share responsibility.

The results include the following:

- Prior to 1996, there was no direct primary care access. In 1996, only 35% of the local Alaska Native population had a designated primary care provider. Of those, 43% did not know who that provider was. Now, more than 95% are empanelled to an integrated primary care team. Providers know their customers’ names, as well as their histories, preferences and family dynamics.
- Before Nuka, the average delay to schedule a routine appointment was 4 weeks. Now, Southcentral Foundation offers same-day access, in person or by phone or email (customer's choice).
- By implementing same-day access, Southcentral Foundation reduced the number of individuals on its behavioral health wait list (backlog) from about 1,300 to nearly zero in a year.
- Phone wait times, before Nuka, were in excess of 2 min, and are now limited to less than 30 s.
- A 36% reduction in hospital days, 42% reduction in ER and urgent care usage, and 58% reduction in specialty clinic visits have been sustained for 10 and above years.
- In 75% of the HEDIS measures (national standards), Southcentral Foundation is in the 75th percentile or better, and for many, like diabetes care, in the 95th percentile.
- Staff turnover is one-fourth of the level it was 5 years earlier.
- 25% increase in childhood immunizations.
- Customer satisfaction with respect for their cultures and traditions at 94%.

Southcentral Foundation has distinguished itself as a role model health care organization. It was Alaska's first health care organization, and 15th health care organization in the nation, to receive the Malcolm Baldrige National Quality Award. The US Congress created this award program in 1987 to identify and recognize the country's most innovative organizations, and then disseminate and share best-practice performance strategies. Southcentral Foundation also achieved the highest level of Patient Centered Medical Home^™^ recognition from the National Committee for Quality Assurance in 2009. The Patient Centered Medical Home standards emphasize the use of systematic, patient-centered, coordinated care that supports access, communication and patient involvement. Southcentral Foundation believes these standards could be improved by focusing on: the individual (in Southcentral Foundation's case, the “customer-owner”) and his/her family driving the system rather than the professionals; services that are woven into customers’ lives built around them, rather than the medical office; and an approach that addresses the whole person and family in a well-coordinated and personal way. A better term for the Patient Centered Medical Home designation might be “customer-driven whole person care” or “customer- and family-driven integrated care provided on their terms.”

Southcentral Foundation's customer-owners recognize that future generations of their families will continue to own, manage and benefit from these services. With this ownership, comes a sense of shared responsibility for the health care system's success. The people of the region are working to continuously improve the services and ensure that the decisions made are in alignment with their needs and values. Consistent with the body of knowledge on community readiness (11), by being involved, Alaska Native people are now more aware of health promotion and disease prevention options and are more interested and willing to make changes.

The value put on relationships in this Alaska Native-owned system of care provides a dramatically different care experience than what was encountered when the health system was under government control. Better relationships have meant not only healthier customer-owners, but also healthier employees and a healthier organization. These outcomes continue to attract health care professionals and government leaders from all over the world who travel far north to Alaska to learn more.
